# Supplementary material for: A systematic review of individual, social, and societal resilience factors in response to societal challenges and crises
Source: Commun Psychol. 2024 Oct 5;2:92. doi: 10.1038/s44271-024-00138-w (PMC11455977; doi:10.1038/s44271-024-00138-w)
Supplement: Supplementary file 2 — Supplementary Information [file 44271_2024_138_MOESM2_ESM.pdf]

Supplementary Material

---

related to '**A systematic review of individual, social, and societal resilience factors in response to societal challenges and crises**'

Sarah K. Schäfer[1,2], Max Supke[1,2], Corinna Kausmann[3], Lea M. Schaubruch[1], Klaus Lieb[1,4], and Caroline Cohrdes[3]

1 Leibniz Institute for Resilience Research, Mainz, Germany
2 Department of Clinical Psychology, Psychotherapy and Psychodiagnostics, Technische Universität Braunschweig, Braunschweig, Germany
3 Department of Epidemiology and Health Monitoring, Robert Koch Institute, Berlin, Germany
4 Department of Psychiatry and Psychotherapy, University Medical Center of Johannes Gutenberg University, Mainz, Germany

Correspondence related to this Supplementary Material to sarah.schaefer@lir-mainz.de.

Table of Contents

Supplementary Note 1. The use of growth mixture modeling in outcome-based resilienc research 2
Supplementary Note 2. Differences between protocol and review (OSF identifier: 10.17605/OSF.IO/GWJVA) 4
Supplementary Note 3. Search strategies per database 8
Supplementary Note 4. Full list of eligible societal challenges and crises 12
Supplementary Note 5. Classification and brief description of all resilience factors identified in the systematic review 13
Supplementary Note 6. Evidence summaries per single outcome type 18
Supplementary Note 7. Evidence from recovery versus less favorable trajectories 28

Supplementary Note 1. The use of growth mixture modeling in outcome-based resilienc research

Resilience is supposed to represent the most common response to stressor exposure, which is among the most prominent statements in resilience research (Galatzer-Levy et al., 2018; Richter-Levin & Sandi, 2021; Roeckner et al., 2021). This notion is largely inspired by the work of George Bonanno (2004) and based on studies applying trajectory-based modeling approaches, most often different variants of growth mixture modeling (GMM; Bryant, 2021). GMM aims at identifying different latent mixture distributions that underly an overall non-normal distribution (Bonanno & Mancini, 2012; Frankfurt et al., 2016). Most of these studies identified 3–5 trajectories (i.e., resilience, recovery, moderate-stable, delayed and chronic responses), with the majority of studies finding resilience to be the most prevalent response (Galatzer-Levy et al., 2018). However, this view has been challenged by the work of Infurna and Luthar (2016b, 2016a) who aimed to replicate previous findings in the field and found the results to largely depend on modeling decisions. In a later review paper (Infurna & Luthar, 2018), they claimed that especially the use of highly restrictive models resulted in high prevalence estimates for resilience. Those restrictive models assume that variances are homogeneous across trajectories and slopes are equal within one trajectory. When using less constrained models, prevalence estimates for resilient responses were decreasing. In response, Galatzer-Levy et al. (2016) stated that the use of less constrained models also reduces their exploratory value.

What are the consequences of this ongoing debate for the current review? It is beyond the scope of this review to present a comprehensive overview on trajectory-based resilience research. In our review, we use individual class assignments as outcomes and examine the predictive value of resilience factors for the (most likely) class membership, with many studies using regression methods also accounting for uncertainty in class assignments (Asparouhov & Muthén, 2014). Thus, there might be some imprecision in our findings resulting from biases in trajectory-based resilience research, yet it is unclear whether this induces rather random error and may thus result in a lack of power or biases our results in a specific direction (i.e., overrating or underrating the relevance of specific resilience factors).

How did we address these methodological issues? Within the current review, we included an assessment of overall model restrictiveness (i.e., fixed variances and slopes) as part of our quality assessment, which was used for sensitivity analysis. As in previous reviews in the field (Schäfer et al., 2022), reporting standards were low for modeling decisions, however, when modeling decisions were not or insufficiently reported, models were rated to be restrictive for a more conservative approach. Additionally, we included the quality of regression modeling, which is key to our review question, as part of our quality assessments, with high-quality studies considering uncertainty in class assignments for regression analysis (Asparouhov & Muthén, 2014).

Supplementary References

Asparouhov, T. & Muthén, B. Auxiliary variables in Mixture Modeling: Three-step approaches using Mplus. *Struct. Equ. Modeling* **21**, 329–341 (2014).

Bonanno, G. A. Loss, trauma, and human resilience: Have we underestimated the human capacity to thrive after extremely aversive events? *Am. Psychol.* **59**, 20–28 (2004).

Bonanno, G. A. & Mancini, A. D. Beyond resilience and PTSD: Mapping the heterogeneity of responses to potential trauma. *Psychol. Trauma* **4**, 74–83 (2012).

Bryant, R. A. A critical review of mechanisms of adaptation to trauma: Implications for early interventions for posttraumatic stress disorder. *Clin. Psychol. Rev.* **85**, 101981; https://doi.org/10.1016/j.cpr.2021.101981 (2021).

Frankfurt, S., Frazier, P., Syed, M. & Jung, K. R. Using Group-Based Trajectory and Growth Mixture Modeling to identify classes of change trajectories. *Couns. Psychol.* **44**, 622–660 (2016).

Galatzer-Levy, I. R. & Bonanno, G. A. It's not so easy to make resilience go away: Commentary on Infurna and Luthar (2016). *Psychol. Sci.* **11**, 195–198 (2016).

Galatzer-Levy, I. R., Huang, S. H. & Bonanno, G. A. Trajectories of resilience and dysfunction following potential trauma: A review and statistical evaluation. *Clin. Psychol. Rev.* **63**, 41–55 (2018).

Infurna, F. J. & Luthar, S. S. Resilience has been and will always be, but rates declared are inevitably suspect: Reply to Galatzer-Levy and Bonanno (2016). *Perspect. Psychol. Sci.* **11**, 199–201 (2016a).

Infurna, F. J. & Luthar, S. S. Resilience to major life stressors is not as common as thought. *Perspect. Psychol. Sci.* **11**, 175–194 (2016b).

Infurna, F. J, & Luthar, S. S. Re-evaluating the notion that resilience is commonplace: A review and distillation of directions for future research, practice, and policy. *Clin. Psychol. Rev.* **65**, 43–56 (2018).

Richter-Levin, G. & Sandi, C. Labels matter: Is it stress or is it trauma? *Transl. Psychiatry* **11**, 385; https://doi.org/10.1038/s41398-021-01514-4 (2021).

Roeckner, A. R., Oliver, K. I., Lebois, L. A. M., van Rooij, S. J. H. & Stevens, J. S. Neural contributors to trauma resilience: A review of longitudinal neuroimaging studies. *Transl. Psychiatry* **11**, 508; https://doi.org/10.1038/s41398-021-01633-y (2021).

Schäfer, S. K., Kunzler, A. M., Kalisch, R., Tüscher, O. & Lieb, K. Trajectories of resilience and mental distress to global major disruptions. *Trends Cogn. Sci.* **26**, 1171–1189 (2022).

Zar, J. H. Spearman rank correlation. In: Encyclopedia of Biostatistics (eds. Armitage, P. & Colton, T.). Chichester, England: John Wiley & Sons. https://doi.org/10.1002/0470011815.b2a15150 (2005).

Supplementary Note 2. Differences between protocol and review (OSF identifier: 10.17605/OSF.IO/GWJVA)

Supplementary Table 1. Differences between protocol and final review

	Protocol	Final review
Theoretical background	no changes	
Review team	no changes	
Searches and search strategies	no changes	
Search update	This update will also include another cluster of stressors (i.e., political and systemic stressors; see Appendix B for details) as well as studies using predictor selection models (e.g., LASSO, Ridge regressions; e.g., Schultebraucks & Galatzer-Levy et al., 2019) for those resilience factors showing the most favourable results within trajectory-based studies.	There were no changes with respect to the search strategy specified in our protocol (and presented below). However, we want to clarify that we did not specifically searched for predictor selection models in databases. Predictor selection models such as LASSO, Ridge regressions were eligible for our analyses, however, we only searched for such models as an alternative for multinomial regression models within primary studies using growth mixture models (GMM) or comparable models to identify differential responses to stressor exposure. However, none of the primary studies eligible for inclusion used predictor selection models.
Review design	Scoping review	The review design has been changed to a systematic review as we were not aware at the date of preregistration that the evidence base will be sufficient to allow for conclusions on specific resilience factors and in-detail analyses. The focus of the current review lies on examining the evidence levels for multilevel resilience factors based on our rating scheme and the (previously specified) focus on identifying research gaps, deriving future research targets and future research questions is less pronounced. Moreover, our work meets all criteria of a systematic review (e.g., quality appraisal). We thus believe that 'systematic review' is a more suitable description of our review.
Review questions	no changes	
Searches and search strategies	no changes	
Eligibility criteria	- Longitudinal observational studies (pre-stressor assessment optional); ≥ 3 time points	No changes have been made, but we added the aspect that the stressor and the start of assessment needed to be at most four years apart. This has been done as studies starting, for example, 10 years after stressor exposure are not suitable to reflect the effects of the stressor. After such a long time, other stressors might have impacted on mental health. Thus, we decided to add this criterion to our eligibility

	Protocol	Final review
		criteria for more straightforward conclusions with respect to stress-related effects.
Eligible outcomes	no changes	
Data extraction	see review protocol	Data extraction as described in the review protocol was further amended by information on effect sizes based on reviewer comments. Specifically, we extracted information from (mostly logistic) regression models and transformed ORs to effect sizes as proposed by Cohen et al. (1988).
Risk of bias (quality) assessment	see review protocol	The quality assessment was further amended by information on trajectory modeling based on reviewer comments. Specially, we coded the restrictiveness of models and whether regression models accounted for uncertainity of class assignments. Both versions of our quality assessment tool are available from the OSF project associated with this review (https://osf.io/9xwyu/).
Data synthesis	Narrative synthesis	no changes
	Rating of evidence levels of resilience factors for resilient outcomes/ trajectories as presented in the review protocol: (see Table 1 and Table 2 below)	The rating scheme has been changed based on data available in the primary studies and reviewer comments. The final version of the rating scheme summarized information from statistical significance and effect sizes.<br>The final rating scheme is presented in the manuscript as well as in the OSF project associated with the review (https://osf.io/9xwyu/), where we also provide a full rating guidance for our scale. Please see below the final version of the rating scheme. (see modified version below)

Table 1. Rating of evidence levels of resilience factors for resilient outcomes/trajectories

Category	Level of evidence (assessed per resilience factor and outcome)
+++	The respective resilience factor is associated with resilient outcomes (i.e., resilient trajectories vs. others or recovery trajectories vs. less favourable trajectories) under control of other resilience factors
++	The respective resilience factor is associated with resilient outcomes (i.e., resilient trajectories vs. others or recovery trajectories vs. less favourable trajectories) under control of sociodemographic variables
+	The respective resilience factor is associated with resilient outcomes (i.e., resilient trajectories vs. others or recovery trajectories vs. less favourable trajectories) without control of other variables
-	The respective resilience factor is not associated with resilient outcomes (i.e., resilient trajectories vs. others or recovery trajectories vs. less favourable trajectories) under control of other resilience factors
--	The respective resilience factor is not associated with resilient outcomes (i.e., resilient trajectories vs. others or recovery trajectories vs. less favourable trajectories) under control of sociodemographic variables
---	The respective resilience factor is not associated with resilient outcomes (i.e., resilient trajectories vs. others or recovery trajectories vs. less favourable trajectories) without control of other variables

Note. Evidence levels ranging from +++ (= highest level of evidence for the respective factor from a single primary study) to --- (= lowest level of evidence for the respective factor from a single primary study).

Table 2. Rating of evidence levels of resilience factors for non-resilient outcomes/trajectories

Category	Level of evidence (assessed per resilience factor and outcome)
+++	The respective resilience factor is not associated with delayed/chronic trajectories or other non-favourable outcomes (i.e., less favourable trajectories vs. resilient/recovery trajectories) without control of other variables
++	The respective resilience factor is not associated with delayed/chronic trajectories or other non-favourable outcomes (i.e., less favourable trajectories vs. resilient/recovery trajectories) under control of sociodemographic variables
+	The respective resilience factor is not associated with delayed/chronic trajectories or other non-favourable outcomes (i.e., less favourable trajectories vs. resilient/recovery trajectories) under control of other resilience factors
-	The respective resilience factor is associated with delayed/chronic trajectories or other non-favourable outcomes (i.e., less favourable trajectories vs. resilient/recovery trajectories) without control of other variables
--	The respective resilience factor is associated delayed/chronic trajectories or other non-favourable outcomes (i.e., less favourable trajectories vs. resilient/recovery trajectories) under control of sociodemographic variables
---	The respective resilience factor is associated with delayed/chronic trajectories or other non-favourable outcomes (i.e., less favourable trajectories vs. resilient/recovery trajectories) under control of other resilience factors

Note. Evidence levels ranging from +++ (= highest level of evidence for the respective factor from a single primary study) to --- (= lowest level of evidence for the respective factor from a single primary study)

Rating of evidence levels of resilience factors – modified version

Category	**OR** (if less favorable trajectory = reference class)	Level of evidence (assessed per resilience factor and outcome)
+++	D) OR ≥ 4.27<br>C) OR ≥ 2.48<br>B) OR ≥ 1.44<br>A) OR < 1.44	The respective resilience factor is significantly associated with resilient outcomes (i.e., resilience trajectories vs. delayed, chronic or other clearly less favorable responses) under control of other resilience factors *and* sociodemographic variables.
++	D) OR ≥ 4.27<br>C) OR ≥ 2.48<br>B) OR ≥ 1.44<br>A) OR < 1.44	The respective resilience factor is significantly associated with resilient outcomes (i.e., resilience trajectories vs. delayed, chronic or other clearly less favorable responses) under control of sociodemographic variables.
+	D) OR ≥ 4.27<br>C) OR ≥ 2.48<br>B) OR ≥ 1.44<br>A) OR < 1.44	The respective resilience factor is significantly associated with resilient outcomes (i.e., resilience trajectories vs. delayed, chronic or other clearly less favorable responses) without control of other variables.
ooo	D) pos.: OR ≥ 4.27<br>C) pos.: OR ≥ 2.48<br>B) pos.: OR ≥ 1.44<br>A) pos.: OR < 1.44	No significant association of the respective resilience factor with any outcomes (i.e., resilience trajectories, recovery trajectories, less favorable trajectories) under control of other resilience factors *and* sociodemographic variables.
oo	D) pos.: OR ≥ 4.27<br>C) pos.: OR ≥ 2.48<br>B) pos.: OR ≥ 1.44<br>A) pos.: OR < 1.44	No significant association of the respective resilience factor with any outcomes (i.e., resilience trajectories, recovery trajectories, less favorable trajectories) under control of sociodemographic variables.
o	D) pos.: OR ≥ 4.27<br>C) pos.: OR ≥ 2.48<br>B) pos.: OR ≥ 1.44<br>A) pos.: OR < 1.44	No significant association of the respective resilience factor with any outcomes (i.e., resilience trajectories, recovery trajectories, less favorable trajectories) without control of other variables.
o	X) mixed (pos. & neg.)	No significant association of the respective resilience factor with any outcomes (i.e., resilience trajectories, recovery trajectories, less favorable trajectories) without control of other variables.
oo	X) mixed (pos. & neg.)	No significant association of the respective resilience factor with any outcomes (i.e., resilience trajectories, recovery trajectories, less favorable trajectories) under control of sociodemographic variables.
ooo	X) mixed (pos. & neg.)	No significant association of the respective resilience factor with any outcomes (i.e., resilience trajectories, recovery trajectories, less favorable trajectories) under control of other resilience factors *and* sociodemographic variables.
o	A) neg.: OR > 0.70<br>B) neg.: OR ≤ 0.70<br>C) neg.: OR ≤ 0.40<br>D) neg.: OR ≤ 0.23	No significant association of the respective resilience factor with any outcomes (i.e., resilience trajectories, recovery trajectories, less favorable trajectories) without control of other variables.
oo	A) neg.: OR > 0.70<br>B) neg.: OR ≤ 0.70<br>C) neg.: OR ≤ 0.40	

	Protocol	Final review

Final review (continued):

	D) neg.: OR ≤ 0.23	No significant association of the respective resilience factor with any outcomes (i.e., resilience trajectories, recovery trajectories, less favorable trajectories) under control of sociodemographic variables.
ooo	A) neg.: OR > 0.70<br>B) neg.: OR ≤ 0.70<br>C) neg.: OR ≤ 0.40<br>D) neg.: OR ≤ 0.23	No significant association of the respective resilience factor with any outcomes (i.e., resilience trajectories, recovery trajectories, less favorable trajectories) under control of other resilience factors *and* sociodemographic variables.
-	A) OR > 0.70<br>B) OR ≤ 0.70<br>C) OR ≤ 0.40<br>D) OR ≤ 0.23	The respective resilience factor is significantly associated with less favorable outcomes (i.e., delayed, chronic or other clearly unfavorable responses vs. resilience trajectories), without control of other variables.
--	A) OR > 0.70<br>B) OR ≤ 0.70<br>C) OR ≤ 0.40<br>D) OR ≤ 0.23	The respective resilience factor is significantly associated with less favorable outcomes (i.e., delayed, chronic or other clearly unfavorable responses vs. resilience trajectories) under control of sociodemographic variables.
---	A) OR > 0.70<br>B) OR ≤ 0.70<br>C) OR ≤ 0.40<br>D) OR ≤ 0.23	The respective resilience factor is significantly associated with less favorable outcomes (i.e., delayed, chronic or other clearly unfavorable responses vs. resilience trajectories) under control of other resilience factors *and* sociodemographic variables.

Note. Evidence levels ranging from +++ (= most favorable level of evidence for the respective factor from a single primary study, i.e., the respective factor showed incremental validity beyond other resilience factors with the highest level of control for other variables) to --- (= least favorable level of evidence for the respective factor from a single primary study, i.e., there is evidence for the respective factor being associated with unfavorable trajectories with the highest level of control for other variables). A = very small association ($d < 0.2$ → OR < 1.44 / > 0.70), B = small effect size / weak association ($d = 0.2$ → OR ≥ 1.44 / ≤ 0.70), C = medium effect size / moderate association ($d = 0.5$ → OR ≥ 2.48 / ≤ 0.40), D = large effect size / strong association ($d = 0.8$ → OR ≥ 4.27 / ≤ 0.23), X = mixed results (positive and negative trends).

Protocol: No statistical program was specified.

Final review: Statistical analyses were performed using *R* version 4.3.2.

Subgroup analyses

Protocol: Clinical (e.g., participants) and methodological (e.g., definition of resilient outcomes, risk of bias) study characteristics that may account for between-study differences will be assessed. Analyses detailed in Table 3 are based on our literature review and preliminary search, they may be adapted during the review process. Analyses will be performed based on qualitative summary tables and we will use non-parametric statistical tests to examine whether specific study, population, and methodological characteristics are associated with higher or lower predictive values of different level resilience factors.

Table 3. Potential variables of interest

**Setting**	• Country of data collection<br>• Region (e.g., North, South, West or Eastern Europe)
**Participants**	• Gender balance per sample<br>• Mean sample age<br>• Previous stressor exposure
**Exposure**	• Stressor type (e.g., natural disaster vs. health stressors vs. war/armed conflicts)
**Methodological aspects**	• Definition of resilient outcomes<br>• Low vs. high reporting standards<br>• Highly restricted vs. medium restricted vs. low restricted growth mixture models
**Study design**	• Prospective vs. longitudinal<br>• Pre-stressor assessment of resilience factors vs. post-stressor assessment of resilience factors

Final review: As stated in our protocol, subgroup analyses were adapted based on available studies per subgroup level. Analyses were performed for:

- Study design (longitudinal vs. prospective)
- Sample mean age
- Gender (im)balance
- Number of assessment waves
- Number of variables/ resilience factors included for modeling
- Aspects of timing (i.e., i) the time interval between the last pre-stressor assessment and occurrence of the stressor; ii) the time interval between stressor exposure and the first post-stressor assessment, and iii) the time interval between stressor exposure and last assessment)
- Stressor type

Analyses used non-parametric statistical tests (as this has been stated in our protocol). These tests were more precisely: Fisher-Freeman-Halton exact tests as equivalent of χ^2 tests with small counts per cell, Kruskal-Wallis tests as non-parametric equivalent of analysis of variances, and Spearman's rank correlations to examine the link between participant characteristics and evidence ratings(Zar, 2005). Tests were selected based on available data.

Data was insufficient for country- or region-level analyses, the impact of previous stressor exposure and pre- vs. post-stressor assessment of resilience factors (however, this was partly reflected in our analyses on prospective vs. longitudinal studies). Between-study differences were too small for

	Protocol	Final review
		definition of resilient outcomes as all studies used a comparable outcome-based approach to resilience. Reporting standards did not allow for the analysis of reporting standards and restrictivness of modeling approaches.
Sensitivity analyses	Were not specified.	We examined the association of effect estimates and NOS ratings using Spearman rank correlations to study the impact of study quality. Moreover, aspects of timing were examined in our sensitivity analyses based on reviewer comments.

Supplementary Note 3. Search strategies per database

For reasons of efficiency, the current review used the search strategy developed for a related project of Thomas et al. (2023). This project assesses a broader range of stressors also including individual-level stressors and positive stressors. Those are not relevant to the current project. Respective records were excluded during the screening processes. The last update of this search was performed on August 2, 2023. This update included the following search terms for all databases (from 2004 to 08/2023): political AND (protest OR activism OR strike) OR ((mistrust OR distrust) AND politics) OR ((mistrust OR distrust) AND media) OR ((societal OR social) AND polari*ation) OR ((societal OR social) AND radicali*ation) OR ((societal OR social) AND (division OR divide)) OR ((violent AND riots OR violent AND clashes) OR poverty OR impoverishment (see here for a documentation of the update: https://osf.io/9xwyu/).

APA PsycNet via EbscoHost (incl. PsycInfo, PsycArticles)

#	Query
1	TI (trauma* OR stress* OR "life event" OR "serious illness" OR "accident" OR "incident" OR "health event" OR "coping" OR "advers*" OR "abuse" OR "violen*" OR expos* OR loss OR disaster OR catastroph* OR "marriage" OR "wedding" OR "parenthood" OR "birth*") OR AB (trauma* OR stress* OR "life event" OR "serious illness" OR "accident" OR "incident" OR "health event" OR "coping" OR "advers*" OR "abuse" OR "violen*" OR expos* OR loss OR disaster OR catastroph* OR "marriage" OR "wedding" OR "parenthood" OR "birth*") OR SU (trauma* OR stress* OR "life event" OR "serious illness" OR "accident" OR "incident" OR "health event" OR "coping" OR "advers*" OR "abuse" OR "violen*" OR expos* OR loss OR disaster OR catastroph* OR "marriage" OR "wedding" OR "parenthood" OR "birth*")
2	TI ("mental health" OR "mental disease" OR "mental distress" OR "mental burden" OR "mental symptoms" OR "psychopatholog*" OR "depress*" OR "anxiety" OR "ptsd" OR "post*traumatic stress" OR "well*being" OR "quality of life" OR "life*satisfaction" OR "grief" OR "post*traumatic growth" OR "resilien*") OR AB ("mental health" OR "mental disease" OR "mental distress" OR "mental burden" OR "mental symptoms" OR "psychopatholog*" OR "depress*" OR "anxiety" OR "ptsd" OR "post*traumatic stress" OR "well*being" OR "quality of life" OR "life*satisfaction" OR "grief" OR "post*traumatic growth" OR "resilien*") OR SU ("mental health" OR "mental disease" OR "mental distress" OR "mental burden" OR "mental symptoms" OR "psychopatholog*" OR "depress*" OR "anxiety" OR "ptsd" OR "post*traumatic stress" OR "well*being" OR "quality of life" OR "life*satisfaction" OR "grief" OR "post*traumatic growth" OR "resilien*")
3	TI ("trajectory" OR "latent growth" OR "latent class" OR "growth mixture" OR "lgmm" OR "gmm" OR "lcgm" OR "lca") OR AB ("trajectory" OR "latent growth" OR "latent class" OR "growth mixture" OR "lgmm" OR "gmm" OR "lcgm" OR "lca") OR SU ("trajectory" OR "latent growth" OR "latent class" OR "growth mixture" OR "lgmm" OR "gmm" OR "lcgm" OR "lca")
4	TI (("prospective" OR "longitudinal" OR "repeated measurement" OR "waves" OR "follow-up")) OR AB (("prospective" OR "longitudinal" OR "repeated measurement" OR "waves" OR "follow-up")) OR SU (("prospective" OR "longitudinal" OR "repeated measurement" OR "waves" OR "follow-up"))
5	#1 AND #2 AND #3 AND #4 (in 2004 – 2023)

PTSDPubs

#	Query
1	title(trauma* OR stress* OR "life event" OR "serious illness" OR "accident" OR "incident" OR "health event" OR "coping" OR "advers*" OR "abuse" OR "violen*" OR expos* OR loss OR disaster OR catastroph* OR "marriage" OR "wedding" OR "parenthood" OR "birth*") OR abstract(trauma* OR stress* OR "life event" OR "serious illness" OR "accident" OR "incident" OR "health event" OR "coping" OR "advers*" OR "abuse" OR "violen*" OR expos* OR loss OR disaster OR catastroph* OR "marriage" OR "wedding" OR "parenthood" OR "birth*") OR mainsubject(trauma* OR stress* OR "life event" OR "serious illness" OR "accident" OR "incident" OR "health event" OR "coping" OR "advers*" OR "abuse" OR "violen*" OR expos* OR loss OR disaster OR catastroph* OR "marriage" OR "wedding" OR "parenthood" OR "birth*")
2	title("mental health" OR "mental disease" OR "mental distress" OR "mental burden" OR "mental symptoms" OR "psychopatholog*" OR "depress*" OR "anxiety" OR "ptsd" OR "post*traumatic stress" OR "well*being" OR "quality of life" OR "life*satisfaction" OR "grief" OR "post*traumatic growth" OR "resilien*") OR abstract("mental health" OR "mental disease" OR "mental distress" OR "mental burden" OR "mental symptoms" OR "psychopatholog*" OR "depress*" OR "anxiety" OR "ptsd" OR "post*traumatic stress" OR "well*being" OR "quality of life" OR "life*satisfaction" OR "grief" OR "post*traumatic growth" OR "resilien*") OR mainsubject("mental health" OR "mental disease" OR "mental distress" OR "mental burden" OR "mental symptoms" OR "psychopatholog*" OR "depress*" OR "anxiety" OR "ptsd" OR "post*traumatic stress" OR "well*being" OR "quality of life" OR "life*satisfaction" OR "grief" OR "post*traumatic growth" OR "resilien*")
3	title("trajectory" OR "latent growth" OR "latent class" OR "growth mixture" OR "lgmm" OR "gmm" OR "lcgm" OR "lca") OR abstract("trajectory" OR "latent growth" OR "latent class" OR "growth mixture" OR "lgmm" OR "gmm" OR "lcgm" OR "lca") OR mainsubject("trajectory" OR "latent growth" OR "latent class" OR "growth mixture" OR "lgmm" OR "gmm" OR "lcgm" OR "lca")
4	title(prospective OR longitudinal OR "repeated measurement" OR waves OR "follow*up") OR abstract(prospective OR longitudinal OR "repeated measurement" OR waves OR "follow*up") OR mainsubject(prospective OR longitudinal OR "repeated measurement" OR waves OR "follow*up")
5	#1 AND #2 AND #3 AND #4 in 2004 - 2023

Full search string: (title(trauma* OR stress* OR "life event" OR "serious illness" OR "accident" OR "incident" OR "health event" OR "coping" OR "advers*" OR "abuse" OR "violen*" OR expos* OR loss OR disaster OR catastroph* OR "marriage" OR "wedding" OR "parenthood" OR "birth*") OR abstract(trauma* OR stress* OR "life event" OR "serious illness" OR "accident" OR "incident" OR "health event" OR "coping" OR "advers*" OR "abuse" OR "violen*" OR expos* OR loss OR disaster OR catastroph* OR "marriage" OR "wedding" OR "parenthood" OR "birth*") OR mainsubject(trauma* OR stress* OR "life event" OR "serious illness" OR "accident" OR "incident" OR "health event" OR "coping" OR "advers*" OR "abuse" OR "violen*" OR expos* OR loss OR disaster OR catastroph* OR "marriage" OR "wedding" OR "parenthood" OR "birth*")) AND (title("mental health" OR "mental disease" OR "mental distress" OR "mental burden" OR "mental symptoms" OR "psychopatholog*" OR "depress*" OR "anxiety" OR "ptsd" OR "post*traumatic stress" OR "well*being" OR "quality of life" OR "life*satisfaction" OR "grief" OR "post*traumatic growth" OR "resilien*") OR abstract("mental health" OR "mental disease" OR "mental distress" OR "mental burden" OR "mental symptoms" OR "psychopatholog*" OR "depress*" OR "anxiety" OR "ptsd" OR "post*traumatic stress" OR "well*being" OR "quality of life" OR "life*satisfaction" OR "grief" OR "post*traumatic growth" OR "resilien*") OR mainsubject("mental health" OR "mental disease" OR "mental distress" OR "mental burden" OR "mental symptoms" OR

"psychopatholog*" OR "depress*" OR "anxiety" OR "ptsd" OR "post*traumatic stress" OR "well*being" OR "quality of life" OR "life*satisfaction" OR "grief" OR "post*traumatic growth" OR "resilien*")) AND (title("trajectory" OR "latent growth" OR "latent class" OR "growth mixture" OR "lgmm" OR "gmm" OR "lcgm" OR "lca") OR abstract("trajectory" OR "latent growth" OR "latent class" OR "growth mixture" OR "lgmm" OR "gmm" OR "lcgm" OR "lca") OR mainsubject("trajectory" OR "latent growth" OR "latent class" OR "growth mixture" OR "lgmm" OR "gmm" OR "lcgm" OR "lca")) AND (title(prospective OR longitudinal OR "repeated measurement" OR waves OR "follow*up") OR abstract(prospective OR longitudinal OR "repeated measurement" OR waves OR "follow*up") OR mainsubject(prospective OR longitudinal OR "repeated measurement" OR waves OR "follow*up"))

Embase.com including PubMed and Medline

#	Query
1	trauma* OR stress* OR 'life event'/exp OR 'life event' OR 'serious illness' OR 'accident'/exp OR 'accident' OR 'incident' OR 'health event' OR 'coping'/exp OR 'coping' OR 'advers*' OR 'abuse'/exp OR 'abuse' OR violen* OR expos* OR 'loss'/exp OR loss OR 'disaster'/exp OR disaster OR catastroph* OR 'marriage'/exp OR 'marriage' OR 'wedding' OR 'parenthood'/exp OR 'parenthood' OR 'birth'/exp OR 'birth*'
2	'mental health'/exp OR 'mental disease'/exp OR 'depression'/exp OR 'depressive symptoms'/exp OR 'anxiety disorder'/exp OR 'anxiety'/exp OR 'posttraumatic stress disorder'/exp OR 'mental stress'/exp OR 'wellbeing'/exp OR 'quality of life'/exp OR 'life satisfaction' OR 'grief'/exp OR 'posttraumatic growth'/exp OR 'resilience'/exp
3	'mental health' OR 'mental disease' OR 'mental distress' OR 'mental burden' OR 'mental symptoms' OR 'psychopatholog*' OR 'depress*' OR 'anxiety' OR 'ptsd' OR 'post*traumatic stress' OR 'well*being' OR 'quality of life' OR 'life*satisfaction' OR 'grief' OR 'post*traumatic growth' OR 'resilien*'
4	#2 OR #3
5	'latent class analysis'/exp OR 'latent class growth analysis'/exp OR 'trajectory analysis'/exp OR 'trajectory'/exp
6	'trajectory' OR 'latent growth' OR 'latent class' OR 'growth mixture' OR 'lgmm' OR 'gmm' OR 'lcgm' OR 'lca'
7	#5 OR #6
8	prospective OR longitudinal OR 'repeated measurement' OR waves OR 'follow*up'
9	'longitudinal study'/exp OR 'prospective study'/exp OR 'follow up'/exp
10	#8 OR #9
11	#1 AND #4 AND #7 AND #10
12	#11 AND (2004:py OR 2005:py OR 2006:py OR 2007:py OR 2008:py OR 2009:py OR 2010:py OR 2011:py OR 2012:py OR 2013:py OR 2014:py OR 2015:py OR 2016:py OR 2017:py OR 2018:py OR 2019:py OR 2020:py OR 2021:py OR 2022:py OR 2023:py)

Scopus.com

#	Query
1	TITLE-ABS-KEY (trauma* OR stress* OR "life event" OR "serious illness" OR "accident" OR "incident" OR "health event" OR "coping" OR "advers*" OR "abuse" OR "violen*" OR expos* OR loss OR disaster OR catastroph* OR "marriage" OR "wedding" OR "parenthood" OR "birth*“)
2	TITLE-ABS-KEY ("mental health" OR "mental disease" OR "mental distress" OR "mental burden" OR "mental symptoms" OR "psychopatholog*" OR "depress*" OR "anxiety" OR "ptsd" OR "post*traumatic stress" OR "well*being" OR "quality of life" OR "life*satisfaction" OR "grief" OR "post*traumatic growth" OR "resilien*")

3	TITLE-ABS-KEY ("trajectory" OR "latent growth" OR "latent class" OR "growth mixture" OR "lgmm" OR "gmm" OR "lcgm" OR "lca")
4	TITLE-ABS-KEY ("prospective" OR "longitudinal" OR "repeated measurement" OR "waves" OR "follow-up")
5	#1 AND #2 AND #3 AND #4 (in 2004 - 2023)

Full search string: (TITLE-ABS-KEY ("prospective" OR "longitudinal" OR "repeated measurement" OR "waves" OR "follow-up")) AND (TITLE-ABS-KEY ("trajectory" OR "latent growth" OR "latent class" OR "growth mixture" OR "lgmm" OR "gmm" OR "lcgm" OR "lca")) AND (TITLE-ABS-KEY ("mental health" OR "mental disease" OR "mental distress" OR "mental burden" OR "mental symptoms" OR "psychopatholog*" OR "depress*" OR "anxiety" OR "ptsd" OR "post*traumatic stress" OR "well*being" OR "quality of life" OR "life*satisfaction" OR "grief" OR "post*traumatic growth" OR "resilien*")) AND (TITLE-ABS-KEY (trauma* OR stress* OR "life event" OR "serious illness" OR "accident" OR "incident" OR "health event" OR "coping" OR "advers*" OR "abuse" OR "violen*" OR expos* OR loss OR disaster OR catastroph* OR "marriage" OR "wedding" OR "parenthood" OR "birth*")) AND (LIMIT-TO (PUBYEAR , 2023) OR LIMIT-TO (PUBYEAR , 2022) OR LIMIT-TO (PUBYEAR , 2021) OR LIMIT-TO (PUBYEAR , 2020) OR LIMIT-TO (PUBYEAR , 2019) OR LIMIT-TO (PUBYEAR , 2018) OR LIMIT-TO (PUBYEAR , 2017) OR LIMIT-TO (PUBYEAR , 2016) OR LIMIT-TO (PUBYEAR , 2015) OR LIMIT-TO (PUBYEAR , 2014) OR LIMIT-TO (PUBYEAR , 2013) OR LIMIT-TO (PUBYEAR , 2012) OR LIMIT-TO (PUBYEAR , 2011) OR LIMIT-TO (PUBYEAR , 2010) OR LIMIT-TO (PUBYEAR , 2009) OR LIMIT-TO (PUBYEAR , 2008) OR LIMIT-TO (PUBYEAR , 2007) OR LIMIT-TO (PUBYEAR , 2006) OR LIMIT-TO (PUBYEAR , 2005) OR LIMIT-TO (PUBYEAR , 2004))

Web of Science

#	Query
1	TS=(trauma* OR stress* OR "life event" OR "serious illness" OR "accident" OR "incident" OR "health event" OR "coping" OR "advers*" OR "abuse" OR "violen*" OR expos* OR loss OR disaster OR catastroph* OR "marriage" OR "wedding" OR "parenthood" OR "birth*")
2	TS=("mental health" OR "mental disease" OR "mental distress" OR "mental burden" OR "mental symptoms" OR "psychopatholog*" OR "depress*" OR "anxiety" OR "ptsd" OR "post*traumatic stress" OR "well*being" OR "quality of life" OR "life*satisfaction" OR "grief" OR "post*traumatic growth" OR "resilien*")
3	TS=("trajectory" OR "latent growth" OR "latent class" OR "growth mixture" OR "lgmm" OR "gmm" OR "lcgm" OR "lca")
4	TS=("prospective" OR "longitudinal" OR "repeated measurement" OR "waves" OR "follow-up")
5	#1 AND #2 AND #3 AND #4
6	#5 and 2023 or 2022 or 2021 or 2020 or 2019 or 2018 or 2017 or 2016 or 2015 or 2014 or 2013 or 2012 or 2011 or 2010 or 2009 or 2008 or 2007 or 2006 or 2005 or 2004 (Publication Years)

Supplementary Note 4. Full list of eligible societal challenges and crises

Populations exposed to all kinds of societal challenges and crises (i.e., societal-level stressors, based on Leppold et al., 2022), such as:

- Pandemics, epidemics (e.g., COVID-19 pandemic, SARS pandemic)
- Conflicts, wars, movements of refugees (e.g., Syrian civil war leading to resettlement in an OECD-country)
- Environmental or natural disasters (e.g., earthquakes, hurricanes, floods, tsunamis, nuclear disasters)
- Climate change
- Non-environmental disasters (e.g., plane crashes, explosions, fires, train accidents, mass panic)
- Terrorist attacks (e.g., 9/11 terrorist attacks)
- Financial or economic crises (e.g., the global financial crisis [GFC])
- Political stressors (e.g., political riots, political activism or protest, mistrust/distrust in politics, mistrust/distrust in media)
- Systemic stressors (e.g., social polarisation, social divide, radicalization, poverty, impoverishment)

Supplementary Reference

Leppold, C., Gibbs, L., Block, K., Reifels, L., & Quinn, P. (2022). Public health implications of multiple disaster exposures. *The Lancet Public Health, 7*(3), e274–e286. https://doi.org/10.1016/S2468-2667(21)00255-3

Supplementary Note 5. Classification and brief description of all resilience factors identified in the systematic review

Individual resilience factors

1. (Cognitive) emotion regulation

Overall emotion regulation (Def.: "conscious thoughts by means of which individuals regulate their emotions in response to stressors"; Schäfer et al., 2023, p. 36)

Emotional clarity (Def.: "the extent to which individuals know (and are clear about) the emotions they are experiencing"; Gratz & Roemer, 2004, p. 47)

*Peaceful disengagemen*t (Def.: "a peaceful attitude toward disengaging the self from the reality"; Kan et al., 2009, p. 305)

Perspective taking (Def.: "the ability to take perspective […] for another person"; Gambin et al., 2020, p. 4)

Positive reframing (Def.: "thinking about negative or challenging situations in a positive way (e.g., thinking about the benefits or upsides of a negative event)"; Schäfer et al., 2023, p. 36)

Tolerance of uncertainty (Def.: tolerance of "the notion that negative events may occur and there is no definitive way of predicting such events"; Carleton et al., 2007, p. 106)

2. Control beliefs

Internal locus of control (Def.: "the belief that outcomes are primarily a result of own action"; Schäfer et al., 2023, p. 36)

Self-efficacy (Def.: an individual's subjective perception of his or her capability to perform a specific behavior or to achieve something; Bandura, 1977)

Sense of mastery (Def.: a person's belief that they are able to control important circumstances in their life; Pearlin & Schooler, 1978)

3. Coping strategies

Overall (adaptive/functional) coping (Def.: a set of intentional, goal-directed efforts to minimize physical, psychological and social harm of stressors; Lazarus & Folkman, 1984)

Active coping (Def.: "the process of taking active steps to try to remove or circumvent the stressor or to ameliorate its effect"; Carver et al., 1989, p. 268)

Coping using emotional support (Def.: "the seeking out of emotional social support, e.g., getting moral support, sympathy, or understanding"; Carver et al., 1989, p. 269)

(Positive) Emotion-focused coping (Def.: coping "aimed at reducing or managing the emotional distress that is associated with (or cued by) the situation"; Carver et al., 1989, p. 267)

Problem-focused coping (Def.: coping "aimed at problem solving or doing something to alter the source of the stress"; Carver et al., 1989, p. 267)

Religious coping (Def.: "the tendency to turn to religion in times of stress"; Carver et al., 1989, p. 270)

Social coping (Def.: "the seeking out of social support"; Carver et al., 1989, p. 269)

4. Dispositional characteristics

Dispositional resilience (Def.: a personality trait that helps individuals to cope with adversity and achieve successful adjustment and development in face of stressors; Hu et al., 2015)

Hardiness (Def.: an ability to handle unexpected changes (challenge) with ease, combined with a sense of meaning in daily life (commitment) and personal control (control); Kobasa, 1979)

Self-reliance (Def.: "e.g., can usually find a way out of difficult situations"; Shilton et al., 2023, p. 4)

Sense of coherence (Def.: “a global orientation that life is comprehensible, manageable, and meaningful”; Schäfer et al., 2023, p. 4)

5. Economic situation

Financial situation | financial stress (inverted) | poverty (inverted) (Def.: (no) economic hardship and unmet expectations of life; Allinson & Berle, 2023; Simich, Hamilton & Baya, 2006)

Individual Income (Def.: the amount of money, property and/or other transfers of value received over a set period of time; often received as exchange for labor or products and can also derive from capital; Merriam-Webster, 2023)

6. Education

Education (in years) (Def.: the process of teaching or acquiring knowledge, skills, and values; VandenBos, 2007; here measured in years spent with education or highest level of education associated with time spent in school)

7. Empathy

Empathy (including empathic concern) (Def.: affective components – resonating with another person´s emotions and being concerned with another person´s distress, cognitive component: taking another person´s perspective; Gladstein, 1983)

8. Flexibility

Cognitive flexibility (Def.: “reflects the ability to update behavior according to different contextual demands”; Hemi et al., 2023, p. 2)

Coping flexibility (Def.: “the ability to engage in different types of coping behaviors”; Bonanno, Pat-Horenczyk & Noll, 2011, p. 118).

Psychological flexibility (“a number of dynamic processes that unfold over time. This could be reflected by how a person: (1) adapts to fluctuating situational demands, (2) reconfigures mental resources, (3) shifts perspective, and (4) balances competing desires, needs, and life domains”; Kashdan & Rottenberg, 2010, p. 2)

9. Meaning and gratitude

Meaning in life | purpose in life (Def.: “the sense made of, and significance felt regarding, the nature of one’s being and existence”; Steger, 2006, p. 81)

Gratitude (Def.: combination of disposition to be grateful for just being; Pellerin, 2022, and a grateful disposition (which is “defined as a generalized tendency to recognize and respond with grateful emotion to the roles of other people’s benevolence in the positive experiences and outcomes that one obtains”; McCullough, Emmons, & Tsang, 2002, p. 112))

10. Positive outcome expectancy

Hope (Def.: “a cognitive set comprising agency (belief in one's capacity to initiate and sustain actions) and pathways (belief in one's capacity to generate routes) to reach goals”; Snyder et al., 1996, p. 321)

Optimism (Def.: the extent to which people hold generalized favorable expectancies for the future; Scheier & Carver, 1985)

11. Religious practice

Religious practice (Def.: the practice of religion often involving behaviors such as prayer, meditation, and participation in collective rituals; VandenBos, 2007)

12. Wisdom

Wisdom (Def.: integration of cognitive, e.g., ability to understand life, reflective, e.g., looking at events from many different perspectives, and affective dimensions, e.g., presence of positive emotions and behavior toward others; Ardelt, 2003)

Social resilience factors

13. Economic situation

Household/family income (also disposable income) (Def.: total income received by all persons living in one household during a set time period; OECD, 2023)

Socioeconomic status (Def.: socioeconomic standing, often measured as a combination of education, profession, and income; VandenBos, 2007)

14. Living situation

Living with family/others (Def.: living with family members, friends, or others in the same household; Fingerman et al., 2021)

15. Partnership

Having a partner (Def.: being in a romantic partnership at the time of the assessment; Merriam-Webster, 2023)

Relationship quality (Def.: positive and negative feelings about the current relationship; Farooqi, 2014)

16. Social integration and participation

Social integration (Def.: extent, to which a person is socially integrated, e.g., interactions with friends, participation in social events; Welch et al., 2016)

Social participation (Def.: "[…] frequency of involvement in community activities"; Raina et al., 2021)

17. Social support

Perceived social support (Def.: the perceived availability and adequacy of social support; Zalta et al., 2021)

Received social support (Def.: the social support actually provided by others; Zalta et al., 2021)

Structural social support (Def.: the size and strength of one's support network; Zalta et al., 2021)

Structural family support (Def.: number of important sources of family support, e.g., parents, children; Shamsikhani et al., 2023)

Structural work support (Def.: number of important sources of work support, e.g., colleagues; Gibson et al., 2022)

Societal resilience factors

18. Collective efficacy

Perceived collective efficacy (Def.: "defined as social cohesion among neighbors combined with their willingness to intervene on behalf of the common good"; Sampson, Raudenbush & Earls, 1997, p. 918)

19. Living environment

Environment quality (Def.: “quality of the physical living environment”; Pellerin et al., 2022, p. 5)

Local house value (Def.: monetary value of the house; Gatzlaff & Haurin, 1998)

Neighborhood environment (Def.: “perceptions of the neighbourhood environment”, e.g., perceived level of trust and safety in neighbourhood; Shilton et al., 2023, p. 4)

Rural region (Def.: “an environment characterized by open land, sparse settlement, some distance from cities and towns”, opposite to living in an urban environment; VandenBos, 2007)

20. Community attachment

Community attachment (Def.: “feeling part of the local community”; Li et al., 2023, p. 3)

21. Climate-related factors

Milder temperature (Def.: Category according to the Australien Building Codes Board, climate zone map - zone 6; https://www.abcb.gov.au/resources/climate-zone-map)

Warmer temperature (Def.: Category according to the Australien Building Codes Board, climate zone map - zone 7; https://www.abcb.gov.au/resources/climate-zone-map)

Supplementary References

Allinson, C. H. & Berle, D. (2023). Association between unmet post-arrival expectations and psychological symptoms in recently arrived refugees. *Transcult. Psychiatry* **60**, 39–51 (2023).

Ardelt, M. Empirical assessment of a three-dimensional wisdom scale. *Research on Aging* **25**, 275-324 (2003).

Bonanno, G. A., Pat-Horenczyk, R. & Noll, J. Coping flexibility and trauma: The perceived ability to cope with trauma (PACT) scale. *Psychol. Trauma **3***, 117–129 (2011).

Bandura, A. (1977). Self-efficacy: Toward a unifying theory of behavioral change. *Psychol. Rev.* **84**, 191–215 (1977).

Carleton, R. N., Norton, M. A. P. J. & Asmundson, G. J. G. Fearing the unknown: A short version of the intolerance of uncertainty scale. *J. of Anxiety Disord.* **21**, 105–117 (2007).

Carver, C. S., Scheier, M. F. & Weintraub, J. K. Assessing coping strategies: A theoretically based approach. *J. Pers. Soc. Psychol.* **56**, 267–283 (1989).

Gambin, M. et al. Factors related to positive experiences in parent–child relationship during the COVID-19 lockdown. The role of empathy, emotion regulation, parenting self-efficacy and social support. PsyArXiv, 1–23. https://doi.org/10.1111/famp.12856 (2020).

Gibson, R. et al. Prevalence and correlates of suicidal ideation in World Trade Center responders: Results from a population-based health monitoring cohort. *J. Affect. Disord.* **306**, 62–70; https://doi.org/10.1016/j.jad.2022.03.011 (2022).

Gladstein, G. A. Understanding empathy: Integrating counseling, developmental, and social psychology perspectives. *J. Couns. Psychol.* **30**, 467–482 (1983).

Gatzlaff, D. H. & Haurin, D. R. Sample selection and biases in local house value indices. *J. Urban Econ.* **43**, 199–222 (1998).

Gratz, K. L. & Roemer, L. Multidimensional assessment of emotion regulation and dysregulation: Development, factor structure, and initial validation of the difficulties in emotion regulation scale. *J. Psychopathol. Behav. Assess.* **26**, 41–54 (2004).

Farooqi, S. The construct of relationship quality. *Journal of Relationships Research* **5**, E2; https://doi.org/10.1017/jrr.2014.2 (2014).

Fingerman, K. L. et al. (2021). Living alone during COVID-19: Social contact and emotional well-being among older adults. *The Journals of Gerontology: Series B* **76**, e116–e121; https://doi.org/10.1093/geronb/gbaa200 (2021).

Hemi, A. et al. Flexibility predicts chronic anxiety and depression during the first year of the COVID-19 pandemic—A longitudinal investigation of mental health trajectories. *Psychol. Trauma* **16**, 961–970 (2024).

Hu, T., Zhang, D. & Wang, J. A Meta-Analysis of the trait resilience and mental health. *Pers. Individ. Differ.* **76**, 18-27 (2015).

Kan, C., Karasawa, M. & Kitayama, S. Minimalist in style: Self, identity, and well-being in Japan. *Self Identity* **8***,* 300–317 (2009).

Kashdan, T. B. & Rottenberg, J. Psychological flexibility as a fundamental aspect of health. *Clin. Psychol. Rev.* **30**, 865–878 (2010).

Kobasa, S. C. Stressful life events, personality, and health: An inquiry into hardiness. *J. Pers. Soc. Psychol.* **37,** 1–11 (1979).

Lazarus, R. S. & Folkman S. (1984). *Stress, appraisal, and coping* (Springer Publishing Company: New York, 1984).

Li, A. et al. Vulnerability and recovery: Long-term mental and physical health trajectories following climate-related disasters. *Soc. Sci. Med.* **320**, 115681; https://doi.org/10.1016/j.socscimed.2023.115681 (2023).

McCullough, M. E., Emmons, R. A. & Tsang, J.-A. The grateful disposition: A conceptual and empirical topography. *J. Pers. Soc. Psychol.* **82**, 112–127 (2002).

OECD. *National Accounts of OECD Countries, Volume 2022 Issue 2*. https://doi.org/10.1787/3e073951-en (2023)
Pearlin, L. I., & Schooler, C. (1978). The structure of coping. *J. Health Soc. Behav.* **19**, 2–21 (1978).
Pellerin, N., Raufaste, E., Corman, M., Teissedre, F. & Dambrun, M. Psychological resources and flexibility predict resilient mental health trajectories during the French COVID-19 lockdown. *Sci. Rep.* **12**, 10674; https://doi.org/10.1038/s41598-022-14572-5 (2022)
Raina, P. et al. A longitudinal analysis of the impact of the COVID-19 pandemic on the mental health of middle-aged and older adults from the Canadian Longitudinal Study on Aging. *Nat. Aging* **1**, 1137–1147 (2021).
Sampson, R. J., Raudenbush, S. W., & Earls, F. Neighborhoods and violent crime: A multilevel study of collective efficacy. *Science* **277**, 918-924 (1997).
Schäfer, S. K. et al. Interrelations of resilience factors and their incremental impact for mental health: insights from network modeling using a prospective study across seven timepoints. *Transl. Psychiatry* **13**, 328; https://doi.org/10.1038/s41398-023-02603-2 (2023).
Scheier, M. F. & Carver, C. S. Optimism, coping, and health: Assessment and implications of generalized outcome expectancies. *Health Psychol.* **4**, 219–247 (1985).
Shamsikhani, S., Ahmadi, F., Kazemnejad, A. & Vaismoradi, M. Design and psychometric properties of the family support for older people questionnaire. *Front. Public Health* **11**, 1068839. https://doi.org/10.3389/fpubh.2023.1068839 (2023)
Shilton, T. et al. Contribution of risk and resilience factors to anxiety trajectories during the early stages of the COVID-19 pandemic: A longitudinal study. *Stress Health* ***39***, 927–939 (2023)
Simich, L., Hamilton, H., & Baya, B.K. Mental distress, economic hardship and expectations of life in Canada among Sudanese newcomers. *Transcult. Psychiatry* **43**, 418-444 (2006).
Snyder, C. R. et al (1996). Development and validation of the state hope scale. *J. Pers. Soc. Psychol.* **70**, 321–335.
Steger, M. F., Frazier, P., Oishi, S. & Kaler, M. (2006). The meaning in life questionnaire: Assessing the presence of and search for meaning in life. *J. Couns. Psychol.* **53***,* 80–93.
Merriam-Webster. Partner. *Merriam-Webster.com dictionary* https://www.merriam-webster.com/dictionary/partner (2023)
Welch, A. E. et al. Trajectories of PTSD among lower Manhattan residents and area workers following the 2001 World Trade Center Disaster, 2003-2012. *J. Trauma. Stress* **29**, 158–166 (2016).
VandenBos, G. R. (Ed.). *APA Dictionary of Psychology* (American Psychological Association: Washington, 2007).
Zalta, A. K. et al. Examining moderators of the relationship between social support and self-reported PTSD symptoms: A meta-analysis. *Psychol. Bull.* **147**, 33–54 (2021).

Supplementary Note 6. Evidence summaries per single outcome type

Anxiety symptoms

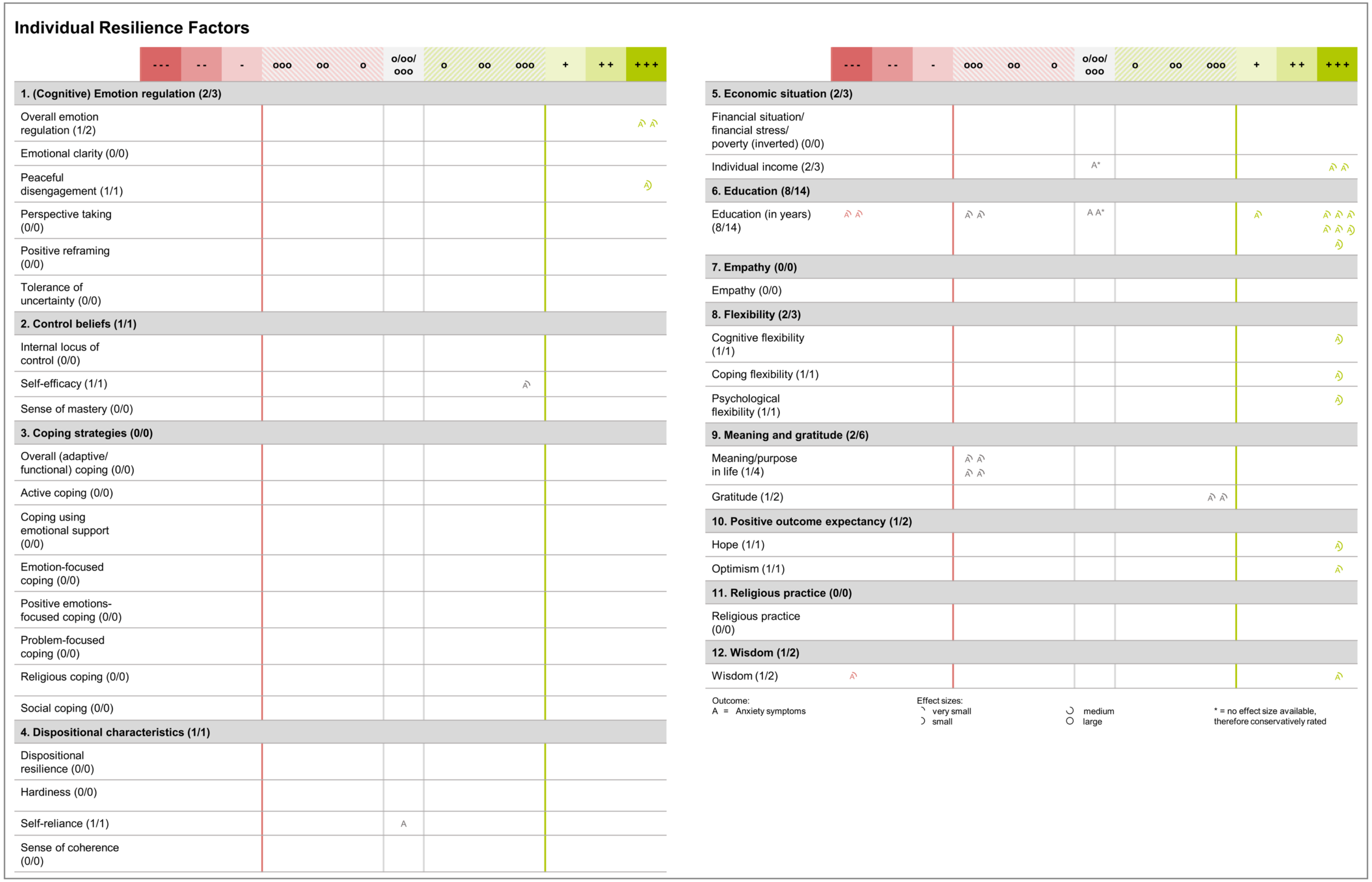

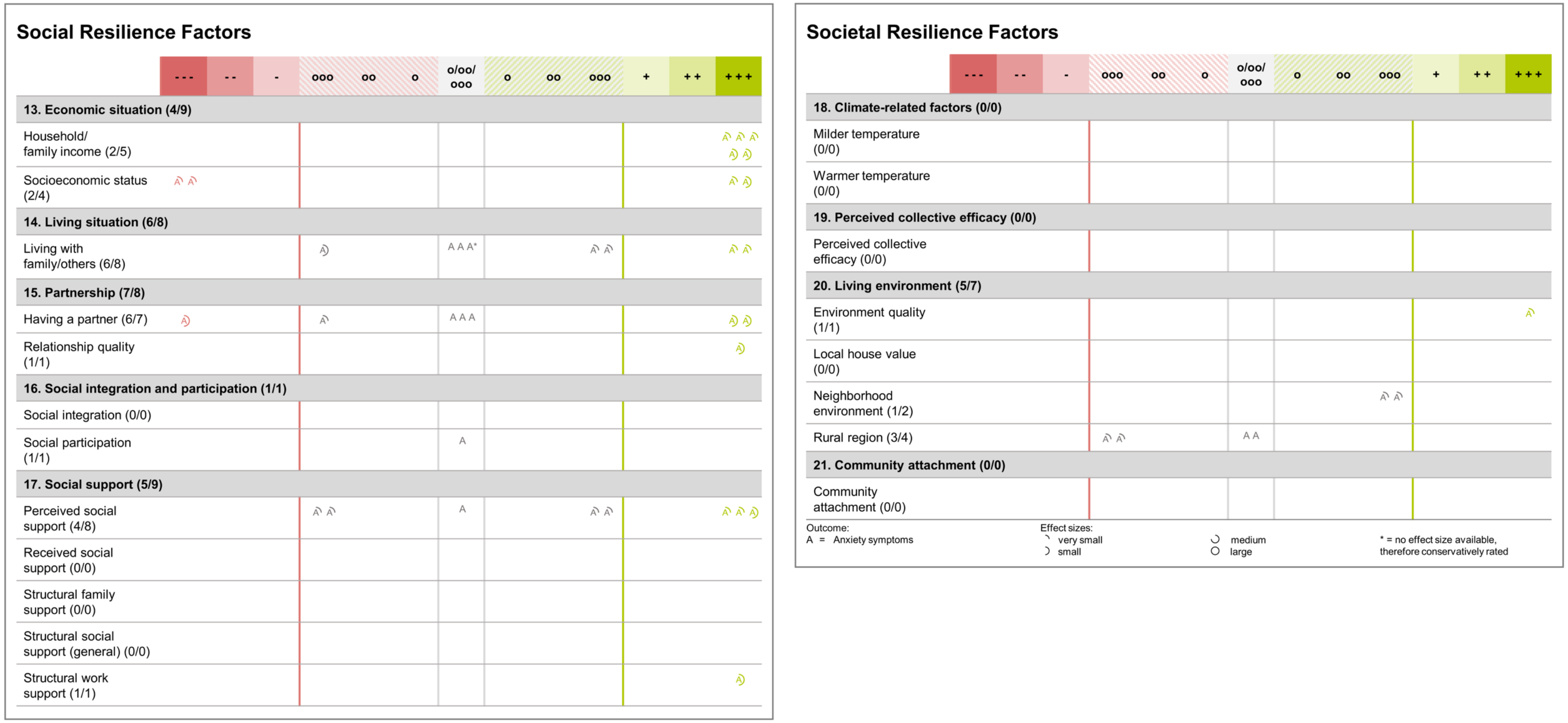

Supplementary Figure 1. Evidence ratings for anxiety symptoms

Depressive symptoms

Individual Resilience Factors

	- - -	- -	-	ooo	oo	o	o/oo/ ooo	o	oo	ooo	+	+ +	+ + +
**1. (Cognitive) Emotion regulation (1/1)**													
Overall emotion regulation (0/0)													
Emotional clarity (0/0)													
Peaceful disengagement (1/1)													D
Perspective taking (0/0)													
Positive reframing (0/0)													
Tolerance of uncertainty (0/0)													
**2. Control beliefs (1/2)**													
Internal locus of control (0/0)													
Self-efficacy (1/2)				D D									
Sense of mastery (0/0)													
**3. Coping strategies (1/3)**													
Overall (adaptive/ functional) coping (1/1)													D
Active coping (0/0)													
Coping using emotional support (0/0)													
Emotion-focused coping (1/1)													D
Positive emotions-focused coping (0/0)													
Problem-focused coping (1/1)				D									
Religious coping (0/0)													
Social coping (0/0)													
**4. Dispositional characteristics (0/0)**													
Dispositional resilience (0/0)													
Hardiness (0/0)													
Self-reliance (0/0)													
Sense of coherence (0/0)													

	- - -	- -	-	ooo	oo	o	o/oo/ ooo	o	oo	ooo	+	+ +	+ + +
**5. Economic situation (3/4)**													
Financial situation/ financial stress/ poverty (inverted) (0/0)													
Individual income (3/4)												D D	D D
**6. Education (13/27)**													
Education (in years) (13/27)		D		D D D D			D D*			D D D D D D D D D D D D D			D D D D D D D
**7. Empathy (0/0)**													
Empathy (0/0)													
**8. Flexibility (2/3)**													
Cognitive flexibility (1/1)													D
Coping flexibility (1/1)													D
Psychological flexibility (1/1)													D
**9. Meaning and gratitude (2/6)**													
Meaning/purpose in life (1/2)							D						D
Gratitude (1/4)				D D D D									
**10. Positive outcome expectancy (1/2)**													
Hope (1/1)							D						
Optimism (1/1)													D
**11. Religious practice (0/0)**													
Religious practice (0/0)													
**12. Wisdom (1/4)**													
Wisdom (1/4)				D D D D									

Outcome:
D = Depressive symptoms

Effect sizes:
◝ very small
◗ small
◡ medium
○ large

* = no effect size available, therefore conservatively rated

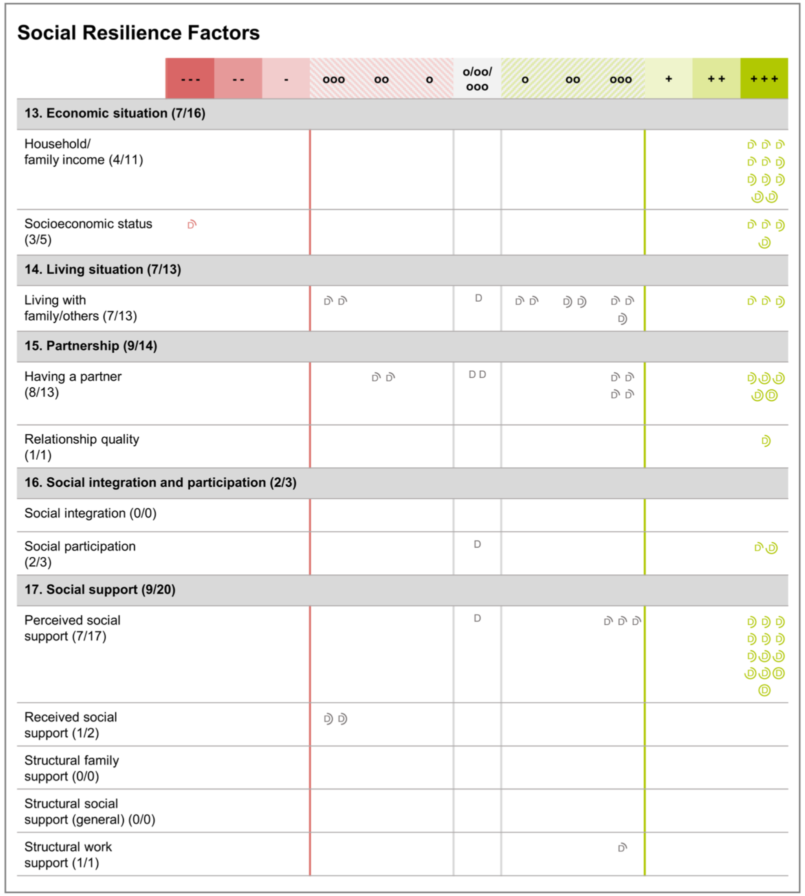

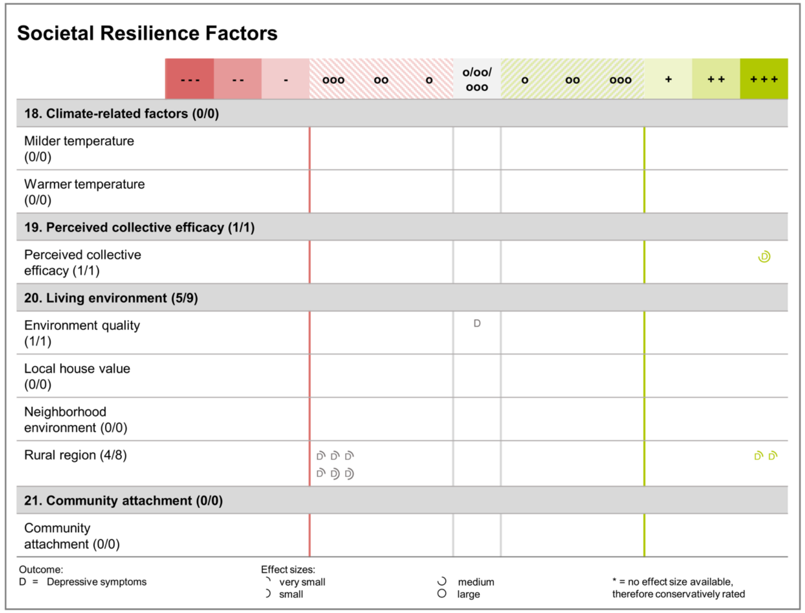

Supplementary Figure 2. Evidence ratings for depressive symptoms

General distress

Individual Resilience Factors

	---	--	-	ooo	oo	o	o/oo/ooo	o	oo	ooo	+	++	+++
**1. (Cognitive) Emotion regulation (3/3)**													
Overall emotion regulation (0/0)													
Emotional clarity (0/0)													
Peaceful disengagement (0/0)													
Perspective taking (1/1)							G						
Positive reframing (1/1)	◔												
Tolerance of uncertainty (1/1)													◔
**2. Control beliefs (3/8)**													
Internal locus of control (3/4)							G						◔ ◔ ◔
Self-efficacy (1/3)				◔ ◔ ◔									
Sense of mastery (1/1)													◔
**3. Coping strategies (2/4)**													
Overall (adaptive/ functional) coping (1/1)													◔
Active coping (1/2)	◔ ◔												
Coping using emotional support (1/1)	◔												
Emotion-focused coping (0/0)													
Positive emotions-focused coping (0/0)													
Problem-focused coping (0/0)													
Religious coping (0/0)													
Social coping (0/0)													
**4. Dispositional characteristics (2/12)**													
Dispositional resilience (2/6)													◔ ◔ ◔ ◔ ◔ ◔
Hardiness (1/3)										◔ ◔ ◔			
Self-reliance (0/0)													
Sense of coherence (1/3)													◔ ◔ ◔

	---	--	-	ooo	oo	o	o/oo/ooo	o	oo	ooo	+	++	+++
**5. Economic situation (6/12)**													
Financial situation/ financial stress/ poverty (inverted) (3/5)													◔ ◔ ◔ ◔ ◔
Individual income (3/7)				◔ ◔									◔ ◔ ◔ ◔ ◔
**6. Education (7/13)**													
Education (in years) (7/13)				◔ ◔			G G G G G						◔ ◔ ◔ ◔ ◔ ◔
**7. Empathy (2/3)**													
Empathy (2/3)	◔									◔ ◔			
**8. Flexibility (0/0)**													
Cognitive flexibility (0/0)													
Coping flexibility (0/0)													
Psychological flexibility (0/0)													
**9. Meaning and gratitude (0/0)**													
Meaning/purpose in life (0/0)													
Gratitude (0/0)													
**10. Positive outcome expectancy (1/1)**													
Hope (0/0)													
Optimism (1/1)							G						
**11. Religious practice (1/1)**													
Religious practice (1/1)							G						
**12. Wisdom (0/0)**													
Wisdom (0/0)													

Outcome:
G = General distress

Effect sizes:
very small
small
medium
large

* = no effect size available, therefore conservatively rated

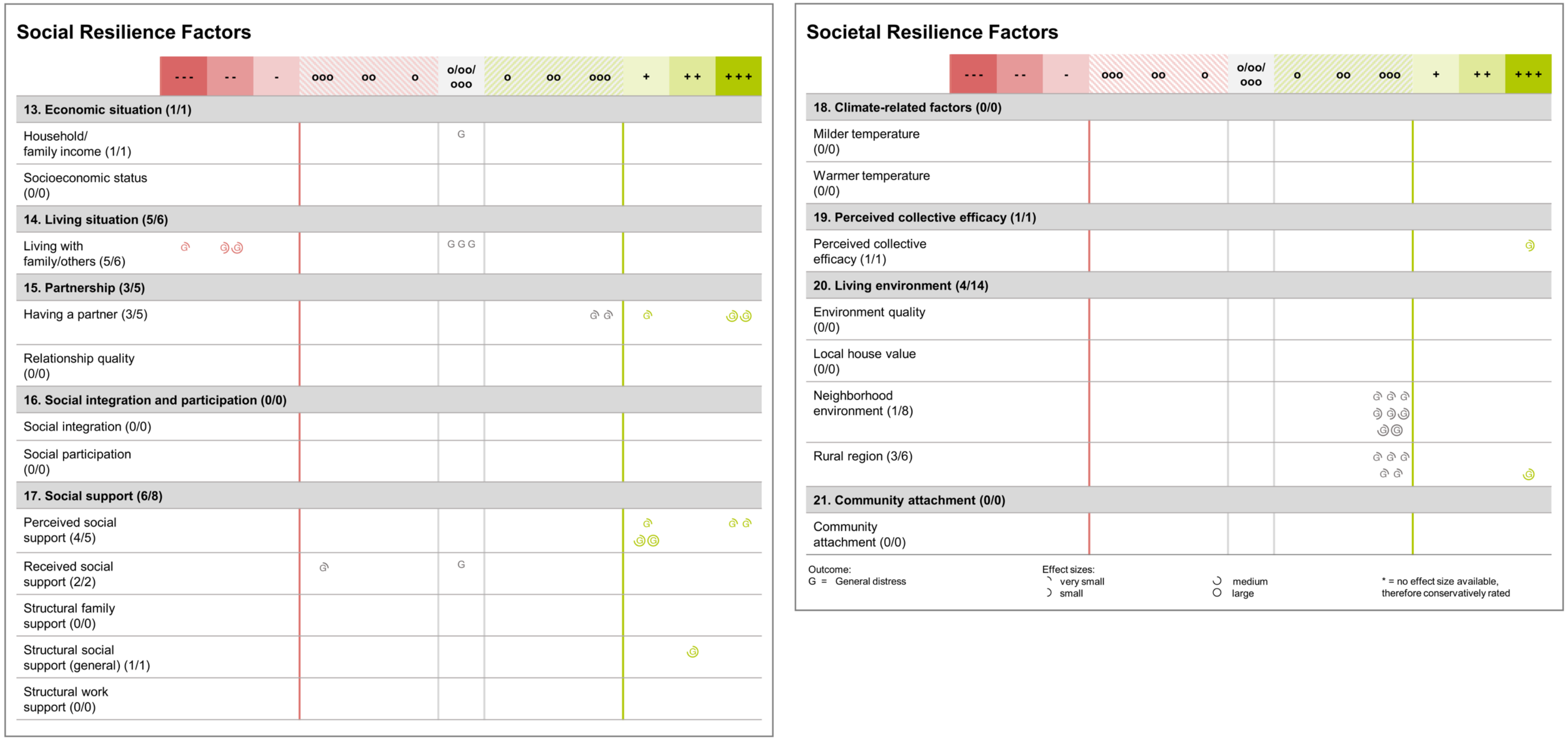

Supplementary Figure 3. Evidence ratings for general distress

Posttraumatic stress

Individual Resilience Factors

	- - -	- -	-	ooo	oo	o	o/oo/ ooo	o	oo	ooo	+	+ +	+ + +
**1. (Cognitive) Emotion regulation (1/4)**													
Overall emotion regulation (1/2)													P P
Emotional clarity (1/2)													P P
Peaceful disengagement (0/0)													
Perspective taking (0/0)													
Positive reframing (0/0)													
Tolerance of uncertainty (0/0)													
**2. Control beliefs (1/1)**													
Internal locus of control (1/1)													P
Self-efficacy (0/0)													
Sense of mastery (0/0)													
**3. Coping strategies (2/15)**													
Overall (adaptive/ functional) coping (1/1)				P									
Active coping (1/2)	P						P						
Coping using emotional support (0/0)													
Emotion-focused coping (0/0)													
Positive emotions-focused coping (1/4)										P P			P P
Problem-focused coping (0/0)													
Religious coping (1/5)	P P P P P												
Social coping (1/3)	P P			P									
**4. Dispositional characteristics (1/3)**													
Dispositional resilience (1/3)										P P P			
Hardiness (0/0)													
Self-reliance (0/0)													
Sense of coherence (0/0)													

	- - -	- -	-	ooo	oo	o	o/oo/ ooo	o	oo	ooo	+	+ +	+ + +
**5. Economic situation (6/12)**													
Financial situation/ financial stress/ poverty (inverted) (1/2)													P P
Individual income (5/10)	P P			P P			P			P P			P P P
**6. Education (9/21)**													
Education (in years) (9/21)	P						P P			P P P P			P P P P P P P P P P P P P P
**7. Empathy (0/0)**													
Empathy (0/0)													
**8. Flexibility (0/0)**													
Cognitive flexibility (0/0)													
Coping flexibility (0/0)													
Psychological flexibility (0/0)													
**9. Meaning and gratitude (2/7)**													
Meaning/purpose in life (2/7)							P P						P P P P P
Gratitude (0/0)													
**10. Positive outcome expectancy (0/0)**													
Hope (0/0)													
Optimism (0/0)													
**11. Religious practice (0/0)**													
Religious practice (0/0)													
**12. Wisdom (0/0)**													
Wisdom (0/0)													

Outcome:
P = PTSD symptoms

Effect sizes:
very small
small
medium
large

* = no effect size available, therefore conservatively rated

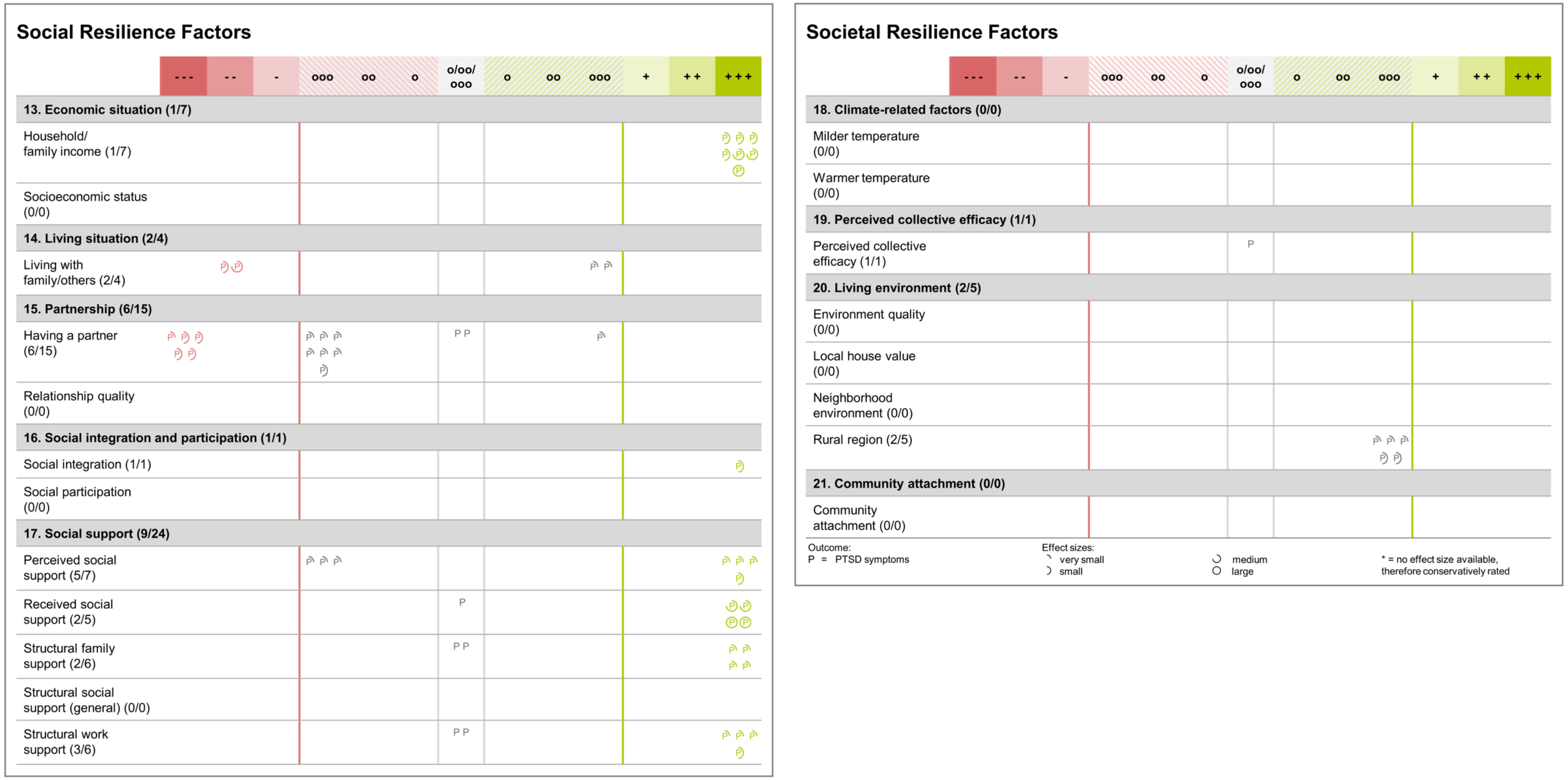

Supplementary Figure 4. Evidence ratings for posttraumatic stress symptoms

Positive mental health outcomes

Individual Resilience Factors

	---	--	-	ooo	oo	o	o/oo/ooo	o	oo	ooo	+	++	+++
**1. (Cognitive) Emotion regulation (1/1)**													
Overall emotion regulation (0/0)													
Emotional clarity (0/0)													
Peaceful disengagement (0/0)													
Perspective taking (0/0)													
Positive reframing (1/1)				O									
Tolerance of uncertainty (0/0)													
**2. Control beliefs (1/3)**													
Internal locus of control (1/1)				O									
Self-efficacy (1/1)										O			
Sense of mastery (1/1)				O									
**3. Coping strategies (2/4)**													
Overall (adaptive/ functional) coping (1/2)													O O
Active coping (1/1)				O									
Coping using emotional support (1/1)				O									
Emotion-focused coping (0/0)													
Positive emotions-focused coping (0/0)													
Problem-focused coping (0/0)													
Religious coping (0/0)													
Social coping (0/0)													
**4. Dispositional characteristics (1/3)**													
Dispositional resilience (1/1)				O									
Hardiness (1/1)							O						
Self-reliance (0/0)													
Sense of coherence (1/1)										O			

	---	--	-	ooo	oo	o	o/oo/ooo	o	oo	ooo	+	++	+++
**5. Economic situation (0/0)**													
Financial situation/ financial stress/ poverty (inverted) (0/0)													
Individual income (0/0)													
**6. Education (3/6)**													
Education (in years) (3/6)	O O O									O			O O
**7. Empathy (0/0)**													
Empathy (0/0)													
**8. Flexibility (0/0)**													
Cognitive flexibility (0/0)													
Coping flexibility (0/0)													
Psychological flexibility (0/0)													
**9. Meaning and gratitude (0/0)**													
Meaning/purpose in life (0/0)													
Gratitude (0/0)													
**10. Positive outcome expectancy (1/1)**													
Hope (0/0)													
Optimism (1/1)	O												
**11. Religious practice (0/0)**													
Religious practice (0/0)													
**12. Wisdom (0/0)**													
Wisdom (0/0)													

Outcome:
O = Positive mental health

Effect sizes:
very small
small
medium
large

* = no effect size available, therefore conservatively rated

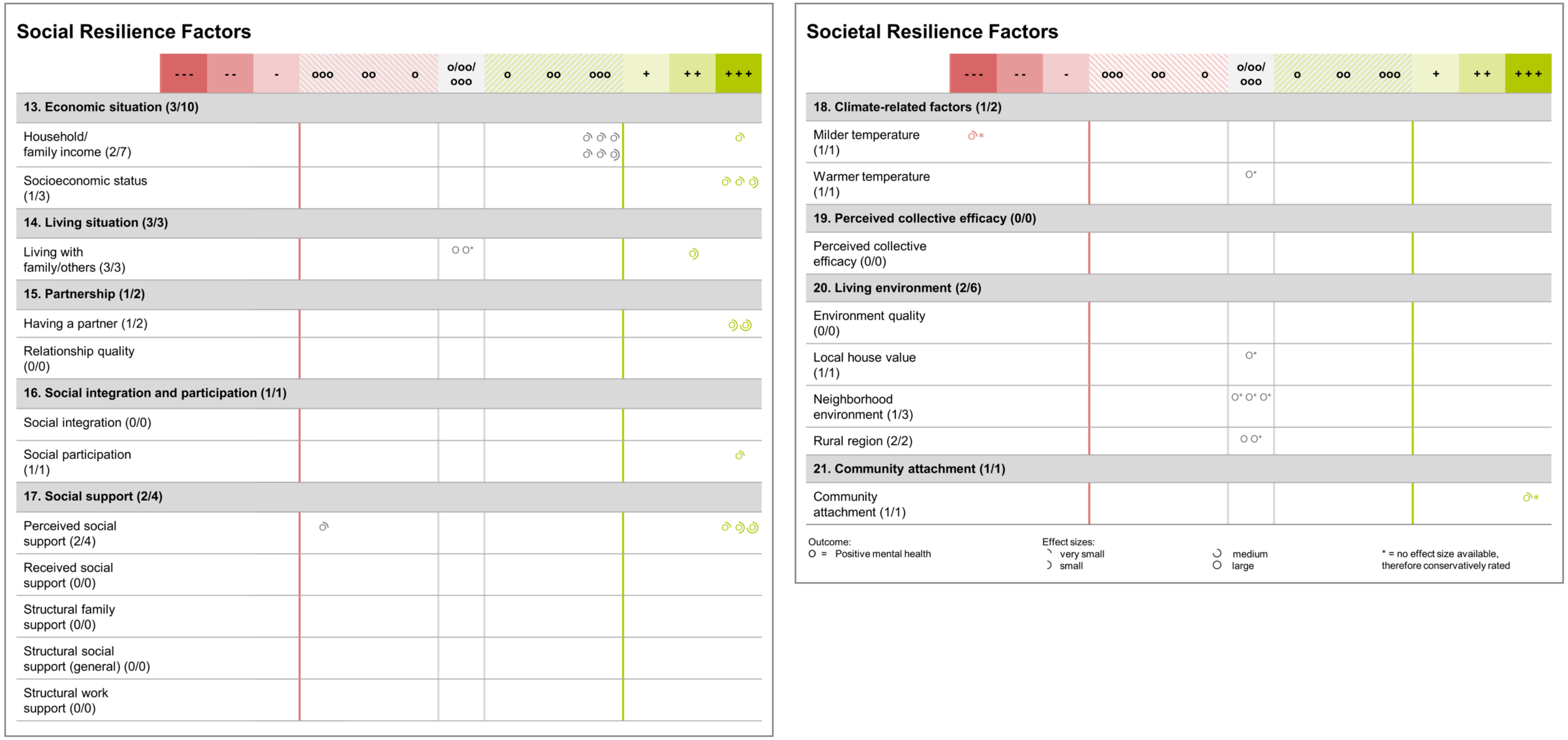

Supplementary Figure 5. Evidence ratings for positive mental health outcomes

Supplementary Note 7. Evidence from recovery versus less favorable trajectories

Individual Resilience Factors

	- - -	- -	-	ooo	oo	o	o/oo/ ooo	o	oo	ooo	+	+ +	+ + +
**1. (Cognitive) Emotion regulation (3/7)**													
Overall emotion regulation (3/3)	G						A P						
Emotional clarity (1/2)										P P			
Peaceful disengagement (0/0)													
Perspective taking (1/2)										G G			
Positive reframing (0/0)													
Tolerance of uncertainty (0/0)													
**2. Control beliefs (0/0)**													
Internal locus of control (0/0)													
Self-efficacy (0/0)													
Sense of mastery (0/0)													
**3. Coping strategies (2/9)**													
Overall (adaptive/ functional) coping (1/1)							P						
Active coping (1/2)										P			P
Coping using emotional support (0/0)													
Emotion-focused coping (0/0)													
Positive emotions-focused coping (1/2)										P			P
Problem-focused coping (0/0)													
Religious coping (1/2)	P									P			
Social coping (1/2)	P			P									
**4. Dispositional characteristics (1/1)**													
Dispositional resilience (0/0)													
Hardiness (0/0)													
Self-reliance (1/1)							A						
Sense of coherence (0/0)													

	- - -	- -	-	ooo	oo	o	o/oo/ ooo	o	oo	ooo	+	+ +	+ + +
**5. Economic situation (6/11)**													
Financial situation/ financial stress/ poverty (inverted) (2/2)													G P
Individual income (4/9)	G G			P			A*			D D P P P			
**6. Education (8/14)**													
Education (in years) (8/14)	G G G						G P			D D A P P P P	A*		P
**7. Empathy (1/2)**													
Empathy (1/2)				G G									
**8. Flexibility (0/0)**													
Cognitive flexibility (0/0)													
Coping flexibility (0/0)													
Psychological flexibility (0/0)													
**9. Meaning and gratitude**													
Meaning in life | purpose in life (1/2)													P P
Gratitude(0/0)													
**10. Positive outcome expectancy (0/0)**													
Hope (0/0)													
Optimism (0/0)													
**11. Religious practice (0/0)**													
Religious practice (0/0)													
**12. Wisdom (0/0)**													
Wisdom (0/0)													

Outcomes: A = Anxiety symptoms; D = Depressive symptoms; G = General distress; O = Positive mental health; P = PTSD symptoms; S = Stress symptoms

Effect sizes: very small; small; medium; large
* = no effect size available, therefore conservatively rated

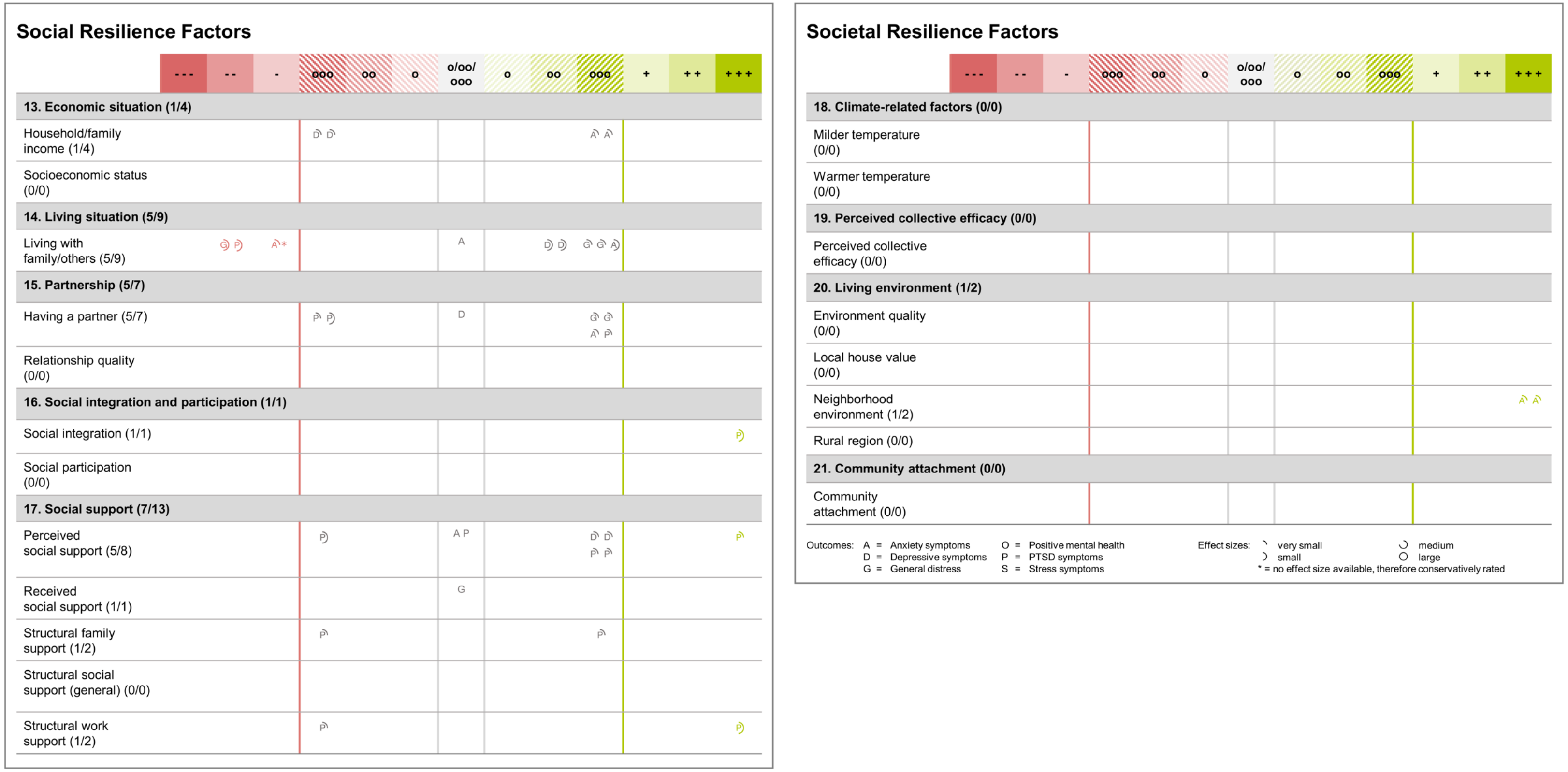

Supplementary Figure 6. Evidence ratings for recovery versus less favorable trajectories
