# Supplementary material for: A systematic review of individual, social, and societal resilience factors in response to societal challenges and crises
Source: Commun Psychol. 2024 Oct 5;2:92. doi: 10.1038/s44271-024-00138-w (PMC11455977; doi:10.1038/s44271-024-00138-w)
Supplement: Supplementary file 3 — Reporting summary [file 44271_2024_138_MOESM3_ESM.pdf]

Corresponding author(s): Sarah K. Schäfer

Last updated by author(s): Aug 26, 2024

Reporting Summary

Nature Portfolio wishes to improve the reproducibility of the work that we publish. This form provides structure for consistency and transparency in reporting. For further information on Nature Portfolio policies, see our Editorial Policies and the Editorial Policy Checklist.

Statistics

For all statistical analyses, confirm that the following items are present in the figure legend, table legend, main text, or Methods section.

n/a	Confirmed	
☒	☐	The exact sample size (*n*) for each experimental group/condition, given as a discrete number and unit of measurement
☐	☒	A statement on whether measurements were taken from distinct samples or whether the same sample was measured repeatedly
☐	☒	The statistical test(s) used AND whether they are one- or two-sided<br>*Only common tests should be described solely by name; describe more complex techniques in the Methods section.*
☐	☒	A description of all covariates tested
☐	☒	A description of any assumptions or corrections, such as tests of normality and adjustment for multiple comparisons
☐	☒	A full description of the statistical parameters including central tendency (e.g. means) or other basic estimates (e.g. regression coefficient) AND variation (e.g. standard deviation) or associated estimates of uncertainty (e.g. confidence intervals)
☒	☐	For null hypothesis testing, the test statistic (e.g. *F*, *t*, *r*) with confidence intervals, effect sizes, degrees of freedom and *P* value noted<br>*Give P values as exact values whenever suitable.*
☒	☐	For Bayesian analysis, information on the choice of priors and Markov chain Monte Carlo settings
☒	☐	For hierarchical and complex designs, identification of the appropriate level for tests and full reporting of outcomes
☐	☒	Estimates of effect sizes (e.g. Cohen's *d*, Pearson's *r*), indicating how they were calculated

Our web collection on statistics for biologists contains articles on many of the points above.

Software and code

Policy information about availability of computer code

Data collection	We used Zotero for de-duplication and Rayyan (Ouzzani, 2016) for title and abstract screening.<br><br>References:<br>Ouzzani, M., Hammady, H., Fedorowicz, Z. & Elmagarmid, A. (2016). Rayyan — a web and mobile app for systematic reviews. Systematic Reviews, 5:210.
Data analysis	Data analyses used IBM SPSS statistics version 29.<br>IBM Corp. Released 2023. IBM SPSS Statistics for Windows, Version 29.0.2.0 Armonk, NY: IBM Corp

For manuscripts utilizing custom algorithms or software that are central to the research but not yet described in published literature, software must be made available to editors and reviewers. We strongly encourage code deposition in a community repository (e.g. GitHub). See the Nature Portfolio guidelines for submitting code & software for further information.

Data

Policy information about availability of data

All manuscripts must include a data availability statement. This statement should provide the following information, where applicable:

- Accession codes, unique identifiers, or web links for publicly available datasets
- A description of any restrictions on data availability
- For clinical datasets or third party data, please ensure that the statement adheres to our policy

Data is available from: https://osf.io/9xwyu/

Research involving human participants, their data, or biological material

Policy information about studies with human participants or human data. See also policy information about sex, gender (identity/presentation), and sexual orientation and race, ethnicity and racism.

Reporting on sex and gender	We differentiate between sex and gender whenever possible. We run analyses on the association between gender distributions and effect estimates for resilience factors. Differential analyses on sex and gender were limited by missing information in primary studies.
Reporting on race, ethnicity, or other socially relevant groupings	We report information from primary studies, however, again, information was missing in many primary studies. We point to potential differences for underrepresented groups.
Population characteristics	We report on the population characteristics of the review sample (p. 13 ff.). We aim at providing comprehensive information. Details on single studies are also provided in Table 1.
Recruitment	NA<br>No participants were recruited. Yet, we assessed recruiting in primary studies.
Ethics oversight	There is no need for ethical approval of systematic reviews at our institutions.

Note that full information on the approval of the study protocol must also be provided in the manuscript.

Field-specific reporting

Please select the one below that is the best fit for your research. If you are not sure, read the appropriate sections before making your selection.

☐ Life sciences ☒ Behavioural & social sciences ☐ Ecological, evolutionary & environmental sciences

For a reference copy of the document with all sections, see nature.com/documents/nr-reporting-summary-flat.pdf

Behavioural & social sciences study design

All studies must disclose on these points even when the disclosure is negative.

Study description	Systematic review ith narrative synthesis informed by non-parametric statistical tests
Research sample	The systematic review included 50 eligible primary stzudies. The studies were performed in 15 solely high-income OECD countries, including USA (18 studies), the United Kingdom (9 studies), and Australia (4 studies).<br>Samples sizes of primary studies ranged between 360 and 65,818 participants. Thirty-seven studies examined adults from the general population, while specific high-risk populations (e.g., healthcare professionals, police staff, migrants, low-income mothers) were examined in 9 studies. Another four studies examined selective subsamples from the general population without particular risk (e.g., university staff, tourists). Only a small share (11 studies) was representative of the respective target population with most using convenience samples. Mean sample age ranged between 20.01 and 78.69 years (weighted mean: 48.58 years), with 13.4% to 100% (weighted mean: 53.05%) of the respondents self-identifying as women. Attrition was insufficiently reported in many studies, but attrition rates were high for most studies (i.e., up to 99%), indicating decreasing data quality over time.
Sampling strategy	The search strategy for this review builds on a larger review project (preregistration-ID: https://doi.org/10.17605/OSF.IO/A9HWN; results will be reported elsewhere). Five databases were searched from 2004 to present (last update: August 2, 2023), including APA PsycNet (incl. PsycInfo, PsycArticles, PsycExtra), Embase (incl. PubMed and EmbaseCore), PTSDPubs, Scopus, and the Web of Science Core Collection. The primary search contained three clusters with search terms related to i) stress exposure (e.g., trauma, stress, life event), ii) mental health (e.g., anxiety, mental distress, wellbeing), and iii) trajectory modelling (e.g., latent growth, trajectory). Terms within one cluster were linked using the Boolean operator OR and clusters were combined using the operator AND. Moreover, reference lists of related systematic reviews and included primary studies were checked for eligible studies.<br>Eligible studies were longitudinal observational studies examining adult individuals (≥ 18 years) from the civil general population, not recruited from military or clinical contexts, who were exposed to all kinds of societal challenges and crises in member countries of the OECD. In line with recent studies in the field of public health, such stressors include pandemics, wars and armed conflicts, the

	climate crisis, and natural disasters. Studies needed to examine trajectories of mental health by means of GMM (or comparable methodological approaches to trajectory modeling aiming at identifying different patterns of mental health over time) and investigate individual, social or societal resilience factors as their predictors (i.e., as an independent variable in a regression analysis). All methods to examine predictor variables were eligible (e.g., three-step and standard multinomial regression analyses). The classification of resilience factors was based on previous reviews n the field and limited to multilevel psychosocial resources. Moreover, some factors (e.g., education, income, family status or socioeconomic status) could either be classified as sociodemographic characteristic or resilience factor. In these cases, variables were included as resilience factors when they were either potentially modifiable by individual or systemic interventions (e.g., education, income) or might provide a proxy measure of rather well-established resilience factors (e.g., family status or living with a partner as indicators of available support). Studies needed to include ≥ 300 participants and to comprise at least three assessment waves, with no requirement for pre-stressor data. However, stressor exposure and the first assessment wave needed to be at most four years apart.
Data collection	We developed a customized data extraction sheet for this review (available from OSF: https://osf.io/9xwyu/). All data of eligible primary studies were extracted by one reviewer and checked by a second, with disagreements being resolved through discussion or consultation of senior team members. Data extraction focused on sample characteristics, types of societal challenges, and trajectories identified using trajectory modeling, and included information needed for later evidence ratings for resilience factors. Moreover, we extracted information needed for later quality appraisal (i.e., representativeness, outcome assessment, statistical model). Data were extracted for the broader outcome categories of mental distress (i.e., general distress, depressive symptoms, anxiety symptoms, posttraumatic stress symptoms, stress symptoms) and positive mental health (i.e., life satisfaction, personal growth, mental health related quality of life, well-being). Resilience factors were classified as either representing individual, social or societal resources by one reviewer, with individual resources being psychological dispositions, beliefs, or capabilities. Social factors were resources that were perceived or available in one's nearer social environment (e.g., family, friends), while societal factors were resources in the wider environment or the whole society (e.g., trust in authorities, legal protection; see Supplementary Note 5 for details on this classification). The decision on resilience factor level was checked by a second reviewer, with all disagreements being discussed and solved in the review team.
Timing	NA
Data exclusions	Studies not meeting the criteria specified in "Sampling strategy".
Non-participation	NA
Randomization	NA

Reporting for specific materials, systems and methods

We require information from authors about some types of materials, experimental systems and methods used in many studies. Here, indicate whether each material, system or method listed is relevant to your study. If you are not sure if a list item applies to your research, read the appropriate section before selecting a response.

Materials & experimental systems

n/a	Involved in the study
☒	☐ Antibodies
☒	☐ Eukaryotic cell lines
☒	☐ Palaeontology and archaeology
☒	☐ Animals and other organisms
☒	☐ Clinical data
☒	☐ Dual use research of concern
☒	☐ Plants

Methods

n/a	Involved in the study
☒	☐ ChIP-seq
☒	☐ Flow cytometry
☒	☐ MRI-based neuroimaging

Plants

Seed stocks	*Report on the source of all seed stocks or other plant material used. If applicable, state the seed stock centre and catalogue number. If plant specimens were collected from the field, describe the collection location, date and sampling procedures.*
Novel plant genotypes	*Describe the methods by which all novel plant genotypes were produced. This includes those generated by transgenic approaches, gene editing, chemical/radiation-based mutagenesis and hybridization. For transgenic lines, describe the transformation method, the number of independent lines analyzed and the generation upon which experiments were performed. For gene-edited lines, describe the editor used, the endogenous sequence targeted for editing, the targeting guide RNA sequence (if applicable) and how the editor was applied.*
Authentication	*Describe any authentication procedures for each seed stock used or novel genotype generated. Describe any experiments used to assess the effect of a mutation and, where applicable, how potential secondary effects (e.g. second site T-DNA insertions, mosiacism, off-target gene editing) were examined.*
